# New York

**Published:** 1895-05

**Authors:** 


### LEGISLATION.

### New York.

The recent effort in the New York State to again extend the time of registration of non-graduates, which was negatived in the Committee, differed in one very material point from a similar bill vetoed at the preceding session of the Legislature by Governor Flower, in that it purposed to admit to register only those who had practised eight years, while the one vetoed required a limit of but three years.

The bill establishing a higher standard of examinations, referred to in our February number has successfully passed the Assembly, and is pending action in the Senate.

The bill exempting veterinarians from jury duty in New York State has passed the Senate by a vote of 32 to none, and the Assembly by 103 to none, and now awaits the Governor's action. Dr. Arthur O'Shea, member of Committee on Legislation, New York State Veterinary Medical Association, and member of Judiciary Committee New York County Veterinary Medical Association, is out in a letter specially requesting the veterinarians of New York State to write the Governor urging his approval of the act. Every veterinarian of New York State should respond to his timely appeal.

A bill has passed the New York Legislature looking to the establishment of a zoölogical garden in New York City. It is to be sincerely desired that it will meet with the Governor's and the Mayor's approval for its great value as an educational factor for the school children of the greater New York. Every veterinarian should lend his aid and encouragement to this proposition.

The bill for the extension of time for registration in New York State has been killed. The bill for the relief of veterinarians from jury duty promises to become a law, though it will need the continued support and watchfulness of the profession throughout the State until it becomes a law.

The Master Horseshoers' bill in the New York State Legislature has not the undivided support of the veterinary profession, and is being opposed strongly by journeyman shoers.

